# A case of sirenomelia associated with atrial septal defect: A rare case report

**DOI:** 10.1016/j.amsu.2022.103626

**Published:** 2022-04-13

**Authors:** Mohamed Abdullahi mohamud, İsmail Gedi İbrahim, Hassan Salad Fidow, Said Abdirahman Ahmed Abdulle

**Affiliations:** a Cardiology Department, Mogadishu Somali Turkey, Recep Tayyip Erdogan Training and Research Hospital, Somalia; b Radiology Department, Mogadishu Somali Turkey, Recep Tayyip Erdogan Training and Research Hospital, Somalia; c Orthopedic Department, Mogadishu Somali Turkey, Recep Tayyip Erdogan Training and Research Hospital, Somalia

**Keywords:** Sirenomelia, Atrial septal defect, Twin patient

## Abstract

**Introduction:**

and importance: Sirenomelia is a life-threatening condition caused by a rare developmental abnormality. According to the research, the incidence of sirenomelia is estimated to be between 1.5 and 4.2 per 100,000 newborns. Around 15% of cases of sirenomelia are related to a twin pregnancy, most commonly in monozygotic cases, with a 7% incidence. We're reporting on a mermaid syndrome case involving twins, one of whom was healthy and the other had sirenomelia.

**Case presentation:**

An 18-year-old female and her first child arrived at the hospital maternity ward, and she had never been there before, and when an ultrasound was performed, it was discovered that she had two babies in her womb and on of them has clung to each other's legs, and a by elective cesarean section was performed to remove the babies, which resulted in the extraction of two boys, one of whom is healthy and the other is clinging to each other's legs.

**Clinical discussion:**

Sirenomelia is a deadly congenital condition that affects the caudal part of the embryonic body. Although the most evident feature is the fusing of the lower limbs, Approximately 49.5 percent of pregnancies are terminated voluntarily due to fetal malformations, according to reports. The abnormality is thought to be caused by a combination of genetic predisposition and a trigger element in the environment, while the exact cause is unknown and thought to be complex. In cases of surviving sirenomelia, treatment can be administered using a multidisciplinary approach.

**Conclusion:**

Mermaid Syndrome is a fatal congenital abnormality with a bleak outlook. Sirenomelia can be diagnosed by ultrasonography. Oligohydramnios and fused lower limbs are important symptoms that aid in diagnosis during the first trimester of pregnancy, with probable termination of the pregnancy indicated if identified early.

## Highlights

• Sirenomelia is a life-threatening condition caused by a rare developmental abnormality.
• Sirenoamlia with an atrial septal defect is rare.
• Interestingly, this case lived 2 weeks.

## 1 Introduction

Sirenomelia is a rare developmental abnormality that makes life difficult. It is characterized by a spectrum of fusions in varying degrees of the lower extremities; agenesis or kidney dysgenesis; lack of external genitalia; imperforate anus; lumbosacral and pelvic bone malformations; and a single umbilical artery are among the symptoms. Cardiac and central nervous system anomalies have also been reported sporadically [1] The mermaid syndrome is an alternate name for sirenomelia because the fused lower extremities in sirenomelia patients resemble mermaids from Greek and Roman mythology, who were represented as having a human's head and upper torso but a dog's tailﬁsh [1] As recorded in the literature, the incidence of sirenomelia is estimated to be approximately between 1.5 and 4.2 per 1,00,000 births. This malformation is more common in male fetuses with a male-to-female ratio of three [1] A twin pregnancy is responsible for approximately 15% of sirenomelia cases, with monozygotic cases accounting for 7% of all cases. When compared to dizygotic twins or singleton pregnancies, the risk of sirenomelia in monozygotic twins is about 100–150 times higher [1]. Classification of sirenomelia in seven types according to Stocker and Heifetz [2] are as follows: type I, all thigh and leg bones are present; type II, single fibula; type III, absent fibula; type IV, partially fused femurs, fused fibulae; type V, partially fused femurs; type VI single femur, single tibia; type VII, single femur, absent tibia. Sirenomelia has been reported to be associated with heart defects such as truncus arteriosus, ventricular septal defect, and patent ductus arteriosus [3] In my research, I have yet to come across any cases of sirenomelia linked to an atrial septal defectin somalia. We present a monozygotic twin pregnancy in which one fetus was diagnosed with sirenomelia and an atrial septal defect, whereas the other twin was perfectly healthy.

## 2 Case report

**Fig. 1:**
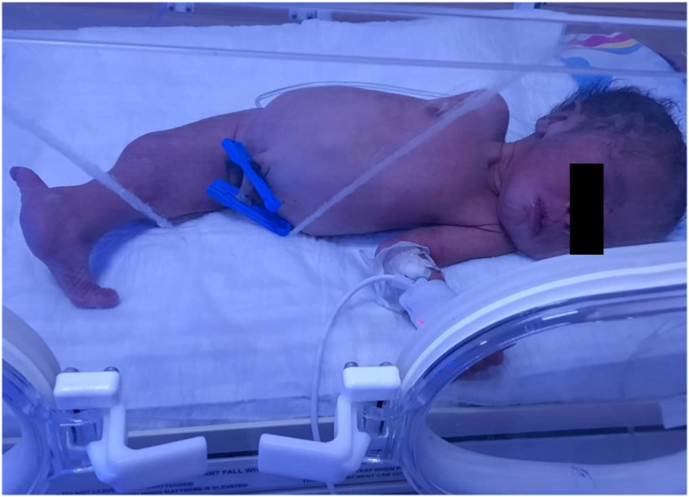
Sirenomelia patient with fused lower limb and two feet.

**Fig. 2:**
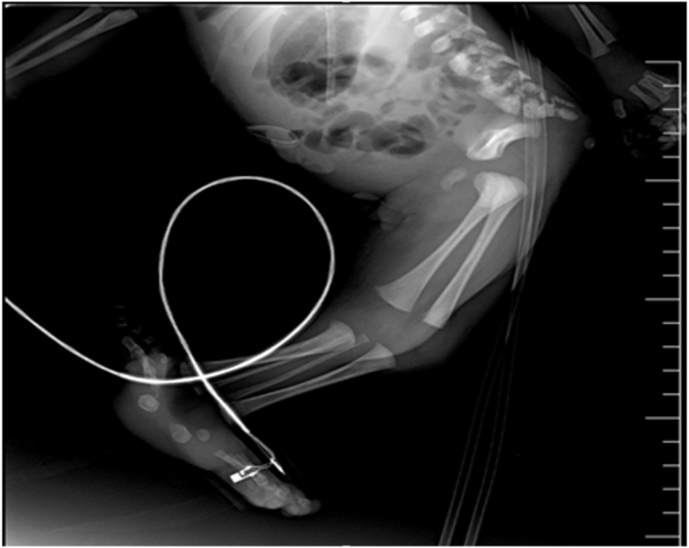
Shows a single lower extremity.

**Fig. 3:**
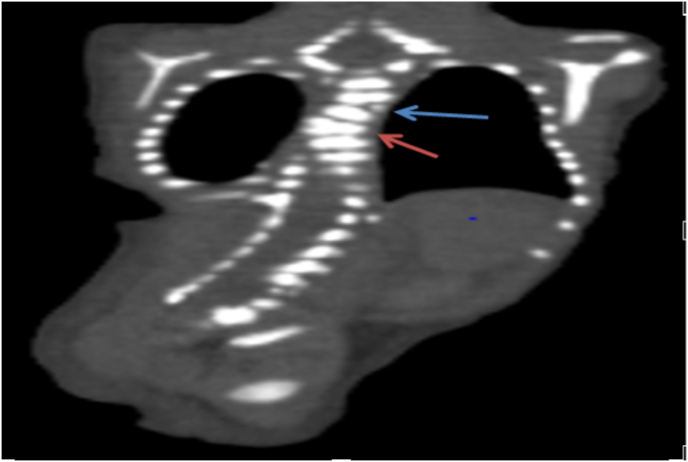
Coronal CT scan shows hemivertebra and scoliosis.

**Fig. 4:**
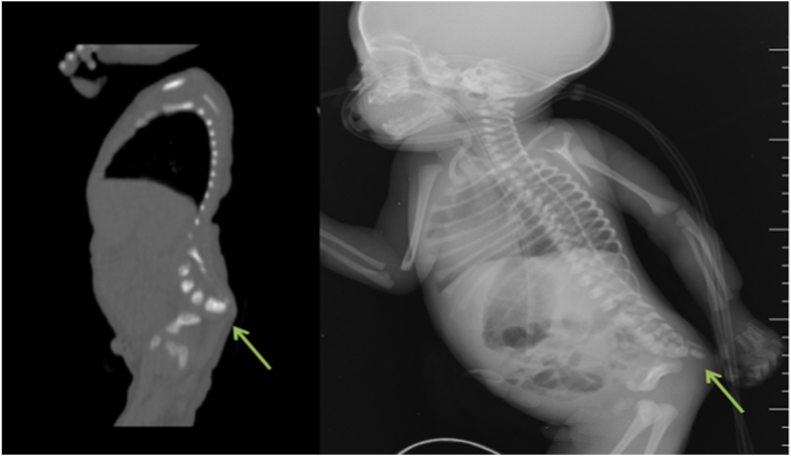
Shows partial sacral agenesis.

**Fig. 5:**
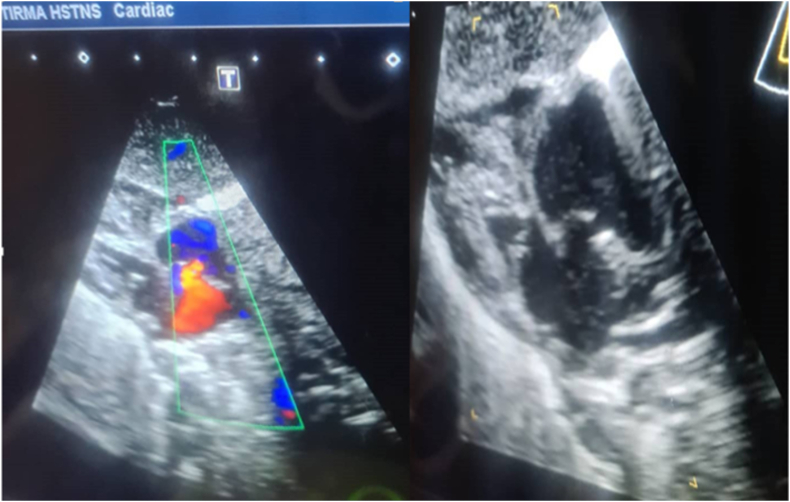
Atrial septal defect.

An 18-year-old female and her first child arrived at the hospital maternity ward, and she had never been there before, but after a medical examination, it was discovered that she had anemia and high blood pressure, and when an ultrasound was performed, it was discovered that she had two babies in her womb and on of them has clung to each other's legs, and a by elective cesarean section was performed to remove the babies, which resulted in the extraction of two boys, one of whom is healthy and the other is clinging to each other's legs. There was no family history of consanguinity between her and her husband. With a weight of 1300 gm and a poor Apgar score, the neonate was born with severe growth restriction and cyanosis. As a result, it was transferred to the newborn intensive care unit shortly after delivery (NICU). Complete fusion of the entire lower limbs with the absence of feet were discovered during a physical examination of the newborn (Fig. 1). There was no anal opening, and no external sexual organs could be seen. The baby was diagnosed with sirenomelia on a clinical level. Radiologic examinations revealed conjoined single lower limbs with two femur, tibia, and fibula (Fig. 2), with Hemivertebra and scoliosis on the middle thoracic vertebral spine (Fig. 3), and sacral agenesis (Fig. 4), all these features compatible with type 1 sirenomelia. Neonatal echocardiography was performed and was diagnosed with the atrial septal defect (Fig. 5). The bladder and both kidneys were not visible on ultrasonography of the abdomen and pelvis, but other abdominal organs appeared normal. Under neonatal care, the neonate lived for two weeks. The other twin was healthy and weighed 2500 g when he was discharged from the hospital after receiving regular preterm care.

## 3 Discussion

The abnormalities seen in sirenomelia are described as the most severe form of caudal regression syndrome [4]. The fusion of the lower limbs, the presence of a single umbilical and persistent vitelline artery are the main features of the Sirtomelia [4]. Although the cause of sirenomelia is unknown, two primary pathogenic hypotheses have been proposed: vascular steal and abnormal blastogenesis [4]. The primary defect in the development of caudal mesoderm is attributed to a teratogenic event during the gastrulation stage. This abnormality prevents the formation of the notochord, resulting in aberrant caudal structure development. Environmental factors include maternal diabetes, tobacco consumption, retinoic acid, and heavy metal exposure [4]. In present case no known environmental variables or teratogenic agent exposure from aetiological factors. Both twins would have been equally exposed to the predisposing factors because there was no history of maternal diabetes or other risk factors and because the co-twin fetus was born healthy. The underlying cause may be an assassination. Sirenomelia is fatal within days of birth due to complications associated with abnormal development and function of the kidneys and urinary bladder. There are around 300 cases recorded worldwide in the literature, 14 of which are from India. In the majority of cases, the diagnosis was made after the baby was born. By using high-resolution or color Doppler sonography in the prenatal stage, sirenomelia can be detected as early as 13 weeks [4]. Similarly our case bladder and both kidneys were not visible on ultrasonography of the abdomen and pelvis, but other abdominal organs appeared normal.

According to the deficient blastogenesis hypothesis, sirenomelia may be considered a severe form of caudal dysgenesis (CD). CD is a diverse group of caudal abnormalities that almost usually include some degree of sacral agenesis [5]. In the presenting case has sacral agenesis.

Sirenomelia carries a very poor prognosis, the affected babies rarely survive beyond 5 days [6]. Because of the typical pulmonary hypoplasia and renal agenesia, sirenomelia is usually fatal. After eight or nine months of pregnancy, approximately 50% of the children are born alive. Death occurs within the first five days after delivery. Interestingly our case lives 2 weeks. A reported one case where a child born with sirenomelia survived; the infant was neurologically normal and had fused lower limbs, imperforate anus, colon atresia, bilateral fused pelvic kidneys with renal dysplasia and sacral dysplasia, and genital anomalies. Laparotomy and colostomy were performed, and the possible separation of the lower limbs was planned (8). Similarly in our case have complete fusion of the entire lower limbs with the absence of feet were discovered during a physical examination of the newborn.

Sirenomelia has been reported to be associated with heart defects such as truncus arteriosus, ventricular septal defect, and patent ductus arteriosus [3] In our case, an atrial septal defect was discovered at 36 weeks of pregnancy, which was later confirmed by neonatal echocardiography. Ours is the first case of sirenomelia associated with an atrial septal defect documented in our country. It is also the first time that sirenomelia has been reported in our hospital. In this case, the etiology of sirenomelia is unknown.

## 4 Conclusion

At 36 weeks of pregnancy, both fetuses were successfully delivered by elective cesarean section, and our case was handled conservatively. The sirenomelic baby lived 2 weeks before dying. It is extremely rare for children with this diagnosis to live longer than three days. The survival of affected newborns is determined by the presence of kidney and renal functions, as well as the severity of the visceral abnormalities. The most common cause of mortality is obstructive renal.

## Ethical approval

Ethical approval for this study was waived by ethical committee of Mogadishu Somali Turkey, Recep Tayyip Erdogan Training and Research Hospital.

## Sources of funding

No funding for this research.

## Author contribution

MAM wrote the manuscript and corrected the manuscript for its scientific basis.

X and SA collected the data for the study.

IGI revised the manuscript for grammar and syntax mistakes.

All authors have read and approved the final manuscript.

## Registration of research studies

1. Name of the registry:

2. Unique Identifying number or registration ID:

3. Hyperlink to your specific registration (must be publicly accessible and will be checked):

## Guarantor

MAM corrected the manuscript for its scientific basis.

## Consent

The Patient's father was invited and written informed consent was obtained for his anonymized information to be published in this study.

## Declaration of competing interest

The authors have no affiliation with any organization with a direct or indirect financial interest in the subject matter discussed in the manuscript.

## References

[1] al Yaqoubi H.N., al Badi M.M., Ambu Saidi F.M., al Shafouri N.S.T. (2018). A case of sirenomelia associated with hypoplastic left heart with a healthy Co-twin: a rare entity. Case Rep. Pediatr..

[2] Khan M., Todase P. (2016 Jun). Sirenomelia: a case Report of a rare congenital anomaly and review of literature. Int. J. Recent Surg. Med. Sci..

[3] Turgut H., Ozdemir R., Gokce I., Karakurt C., Karadag A. (2017). Sirenomelia associated with the hypoplastic left heart in a newborn. Balkan J. Med. Genet..

[4] Samal S.K., Rathod S. (2015 Jan 1). Sirenomelia: the mermaid syndrome: Report of two cases. J. Nat. Sci. Biol. Med..

[5] Garrido-Allepuz C., González-Lamuño D., Ros M.A. (2012 Sep 17). Sirenomelia phenotype in Bmp7;Shh compound mutants: a novel experimental model for studies of caudal body malformations. PLoS One.

[6] Fadhlaoui A., Khrouf M., soumaya Gaigi, Zhioua F., Chaker A. (2010). The sirenomelia sequence: a case history [Internet]. Clin. Med. Insights: Case Rep..

